# Trauma-focused treatments for refugee children: study protocol for a randomized controlled trial of the effectiveness of KIDNET versus EMDR therapy versus a waitlist control group (KIEM)

**DOI:** 10.1186/s13063-022-06178-z

**Published:** 2022-04-23

**Authors:** Merel E. Velu, Irene Martens, Mona Shahab, Carlijn de Roos, Ruud A. Jongedijk, Michaela Schok, T. Mooren

**Affiliations:** 1 ARQ Centrum’45, partner in ARQ National Psychotrauma Centre, Diemen, The Netherlands; 2 Department of Clinical Psychology, Utrecht University, Utrecht, The Netherlands; 3 ARQ Centrum’45, partner in ARQ National Psychotrauma Centre, Oegstgeest, The Netherlands; 4 Department of Clinical Psychology, Leiden University, Leiden, The Netherlands; 5 Department of Epidemiology, Leiden University Medical Center, Leiden, The Netherlands; 6 King Salman Center for Disability Research, Riyadh, Saudi Arabia; 7 Department of Child and Adolescent Psychiatry, Amsterdam University Medical Centre, Amsterdam, the Netherlands

**Keywords:** Psychotrauma, EMDR, KIDNET, Therapy, Refugees, Children, Posttraumatic stress disorder, Randomized controlled trial

## Abstract

**Background:**

Prevalence of posttraumatic stress disorder (PTSD) in refugees is reportedly higher in comparison to the general population. Refugee children specifically are often coping with trauma and loss and are at risk for mental health difficulties. With staggering numbers of people seeking refuge around the world and 50% being 18 years or younger, research examining the effects of trauma-focused therapies for refugee children with PTSD is highly needed. Both Eye Movement Desensitization and Reprocessing (EMDR) therapy and the child version of Narrative Exposure Therapy (KIDNET) have been used for refugees, although these treatment methods have not been systematically compared. The aim of the current study is to investigate the effectiveness of EMDR and KIDNET, compared to a waitlist control group and with each other, offered to refugee children.

**Methods:**

A randomized controlled three-arm trial has been designed. The primary outcome is PTSD diagnosis and symptom severity assessed with the Clinician-Administered PTSD Scale for Children DSM5 (CAPS-CA-5) at baseline (T1), 1 month post-treatment, or after 8 weeks of waiting (T2) and 3 months follow-up (T3). Additionally, instruments to assess posttraumatic stress symptoms, behavioral and emotional problems, and quality of life perception in children aged 8–18 are conducted at T1, T2, and T3.

**Discussion:**

This is the first RCT that examines the effectiveness of EMDR and KIDNET in refugee children aged 8–18 years specifically, compared to a waitlist control group intended to reduce PTSD diagnosis and severity of posttraumatic stress symptoms and comorbid complaints in a growing and challenging population.

**Trial registration:**

Dutch Trial Register NL40769. Retrospectively registered on June 16, 2021.

## Background and rationale

In 2015, European countries faced unusually high arrivals of more than 1.2 million asylum seekers [1]. About half of this population consists of minors. Many of them have been exposed to multiple stressful experiences, such as war, violence, separation, and migration, and are facing continuing challenging living circumstances and uncertainty [2, 3]. At the same time, they often have parents undergoing similar or higher levels of stress. Parental stress has been associated with an increase of psychosocial problems, anxiety and depression symptoms in children, and attachment-related difficulties in infants [4, 5].

Although refugee children and adolescents show impressive resilience and flexibility, they are highly vulnerable to the effect of prolonged traumatic stress due to their age and developmental stage [6]. Despite variations in prevalence rates, there is a consensus that mental health problems, such as PTSD, are higher in refugee children than in general populations [7]. A recent systematic review reports prevalence rates of posttraumatic stress disorder (PTSD) between 19.0 and 52.7% among young refugees and asylum seekers in European countries [8]. Untreated PTSD can lead to the development of anxiety disorders such as generalized anxiety disorder, panic disorder, or mood disorders such as depression or substance use disorders [9]. Ultimately, children’s personality development may be affected by early traumatization. To prevent these harmful long-term effects, effective and timely treatment of PTSD is necessary.

While increasing studies evaluate the effectiveness of psychological interventions for traumatized children [10, 11], hardly any attention is being devoted to the application of interventions to traumatized refugee children specifically. Several studies on the effects of trauma-focused cognitive behavioral therapy (TF-CBT) report a decrease in trauma-related symptoms in children from war-affected countries after TF-CBT, compared to a control group [12–15]. Although results are promising, intervention studies focusing on traumatized refugee children are not only scarce, but also often methodologically weak [16]. As traumatized refugee children and adolescents are increasingly being identified as a specific group within the mental health field and their number is growing, it is needed to shed more light on the effects of trauma-focused treatment for this population. In this paper, a randomized controlled trial (RCT) is described focusing on the effectiveness of two trauma-focused treatments, KIDNET and EMDR, compared to a waitlist control group in reducing PTSD symptoms and comorbid symptoms in refugee children. In Tables 1 and 2, an overview of the studies on EMDR and KIDNET in refugee children is presented.

**Table 1:** Studies on the effectivity of (KID)NET with refugee children

Study	Studied population	*N*	Study design	Treatment	Number and duration of sessions	Instrument PTSD	Measure points	Results
Catani et al. (2009) [17]	Internally displaced children aged 8–14 in Sri Lanka	31	RCT	KIDNET vs MED-RELAX	6 sessions of 60–90 min	UPID	Pretreatment and 1-month and 6-month follow-up	Significant reduction of PTSD symptoms in both conditions, no significant differences between conditions were found. Effect sizes (Cohen’s *d*) for the KIDNET group were 1.76 at post-test and 1.96 at 6 months follow-up.
Ertl et al. (2011) [18]	Former child soldiers aged 12–25 in Uganda	85	RCT	(KID)NET vs academic catch-up program vs waitlist	8 sessions of 90–120 min	CAPS, revised version for DSM-IV	Pretreatment and 3-month, 6-month, and 12-month follow-up	Significant reduction of PTSD symptoms, superiority of (KID)NET (Cohen’s *d* = 1.80).
Onyut et al. (2005) [19]	Refugee children aged 13–17 from Somali in Uganda	6	Pre-post study	KIDNET	4–6 sessions of 1–2 h	CIDI (PDS + HSCL for screening)	Pre-treatment, 4 weeks and 9-month follow-up	After 9 months, four of the six participants no longer met the criteria for PTSD.
Peltonen et al. (2019) [20]	Refugee children and children with experiences of family violence aged 9–17 living in Finland	50	RCT	KIDNET vs TAU	7–10 sessions of 90 min	CRIES-13	pre-, mid-, and posttreatment and 3-month follow-up	No evidence was found for superior effects of NET versus TAU on reduction in PTSD symptoms. There was a decrease in PTSD symptoms regardless of treatment condition; however, this decrease was significant in the NET group only. The effect sizes (Cohen’s *d*) were large in NET (0.83), but small in TAU (0.37)
Ruf et al. (2010) [21]	Refugee children aged 7–16 in Germany	26	RCT	KIDNET vs waitlist	7–8 sessions of 90–120 min	UPID	Pretreatment, 4-weeks, 6-month, and 12-month follow-up	The KIDNET-group (ES=1.9), but not the waitlist (ES=0.3), showed clinically relevant and significant reduction in PTSD symptoms.
Said et al. (2020) [22]	UCM aged 16–17 from Sudan, Vietnam, and Albania in the UK	4	Pre-post study	KIDNET	9–20 sessions	CRIES-8 + CPSS-5	pre-, start-, mid-, and posttreatment	PTSD symptoms were below the clinical range after treatment. All three participants who completed KIDNET met the criteria for reliable improvement.
Schauer et al. (2004) [23]	Refugee child aged 13 from Somalia in Uganda	1	Case study	KID-NET	4 sessions of 60–90 min	PDS	Pretreatment and at 6-month follow-up	The post-test showed that the symptoms decreased to a degree below the diagnostic threshold for PTSD.

Note: *UPID* UCLA PTSD index for DSM-IV, *CIDI* Composite International Diagnostic Interview, *PDS* The Posttraumatic Diagnostic Scale, *HSCL* the Hopkins Symptom Checklist-25, *CRIES* Child Revised Impact of Events Scale, *CPSS-5* Child PTSD Symptom Scale

**Table 2:** Studies on Effectivity of EMDR With Refugee Children

Study	Studied population	N	Study design	Treatment	Number and duration of sessions	Instrument PTSD	Measure points	Results
Oras et al. (2004) [24]	Refugee children aged 8–16 in Sweden	13	Pre-post study	Age 8–13: EMDR + play therapy Age 13+: EMDR + conversational therapy	Total 5–25 sessions of which 1–6 sessions of EMDR	PTSS-C, GAF	Pre- and posttreatment	A significant improvement was found in the functioning level and all PTSS-C scales. The improvement in the functioning level was significantly correlated with the reduction of the PTSD-non-related and the depression symptoms, but not with the PTSD-related symptoms.
Wadaa et al. (2010) [25]	Refugee children aged 7–12 from Iraq in Malaysia	37	(Nonrandom) controlled trial	EMDR vs no treatment	12 sessions	UPID	Pre- and posttreatment	EMDR, but not the control group, was effective in reducing PTSD symptoms. (Hedges’s *g* = 3.46*)

Note: *PTSS-C* The Posttraumatic Stress Symptom Scale for Children, *GAF* Global Assessment of Functioning, *UPID* UCLA PTSD index for DSM-IV

*Effect size calculated by author

### Child version of Narrative Exposure Therapy (KIDNET)

Narrative Exposure Therapy (NET), originally developed in the context of a refugee camp [26, 27], is an evidence-based trauma treatment developed especially for multiple traumatized people exposed to repeated and various traumatic stressors, such as war, torture, rape, childhood abuse, and other forms of multiple and organized violence [28]. While initially designed for adults, adaptations were made from the start for the use of NET in children 8 years and older: KIDNET [19, 23]. KIDNET is focused on reducing posttraumatic stress symptoms by the technique of narrative exposure. By working through the child’s biography and focusing on the autobiographical elaboration of traumatic experiences, KIDNET facilitates processing traumatic memories of traumatic experiences from the child’s recent past [29]. The effectiveness of NET in reducing PTSD symptoms in adults has been established in several randomized controlled trials and several studies show promising results regarding the effectiveness of KIDNET in treating children with PTSD [30, 31].

Seven studies evaluated the effects of KIDNET in treating refugee children with PTSD symptoms (Table 1). Promising evidence regarding the effectiveness of KIDNET in refugee children with PTSD symptoms has been found in four RCTs, two pre- to posttest studies, and one case study [17–23]. In all studies, a reduction of PTSD symptoms was found after receiving KIDNET. KIDNET was compared to meditation-relaxation, treatment as usual (TAU), a waitlist control group, and with an academic catch-up program. The number of participants in the studies ranged from 1 to 85. Some limitations of the study designs included a lack of a control group in some studies, a small sample size, and the absence of a clinical interview in most studies. The CAPS-CA, seen as the golden standard for measuring PTSD in children, has been only administered in one study.

### Eye Movement Desensitization Reprocessing (EMDR)

EMDR is an evidence-based trauma treatment for processing traumatic memories in children and adults [32]. The core feature of EMDR is that an individual recalls a distressing memory while also performing a secondary task (dual taxation), usually engaging in sets of saccadic eye movements. Both tasks compete for limited resources of the working memory. As a consequence, the distressing memory becomes less vivid and emotional [33]. The effectiveness of EMDR in reducing PTSD symptoms in adults has been established in several randomized controlled trials [34]. Moreover, many studies show promising results regarding the effectiveness of EMDR in treating children with PTSD [35]. While EMDR has been recommended by international guidelines, only two studies have been known to us that assess the effects of individual EMDR treatment in refugee children with PTSD symptoms (Table 2). The results of the two studies demonstrated significant improvement on PTSD symptoms [24, 25]. However, serious disadvantages to the study designs included a small sample size, the lack of randomization, lack of self-report instruments, restricted age range of participants, and possible bias in assigning children to the treatment conditions (unequal subsample sizes).

### Objectives of the current trial

The aim of the current study is to investigate the efficacy of both EMDR and KIDNET compared to a waitlist control group. The research questions are:

1. Is EMDR effective in decreasing PTSD and posttraumatic stress symptoms, decreasing behavioral and emotional symptoms, and improving quality of life in refugee children and adolescents, compared to a waitlist control group?

We hypothesize that EMDR is more effective in reducing PTSD and posttraumatic stress symptoms, behavioral and emotional symptoms, and improving quality of life in refugee children and adolescents, compared to a waitlist control group.

2) Is KIDNET effective in decreasing PTSD and posttraumatic stress symptoms, decreasing behavioral and emotional symptoms, and improving quality of life in refugee children and adolescents compared to a waitlist control group?

We hypothesize that KIDNET is more effective in reducing PTSD and posttraumatic stress symptoms, behavioral and emotional symptoms, and improving quality of life in refugee children and adolescents, compared to a waitlist control group.

3) Is there a difference in effectivity between EMDR and KIDNET?

We hypothesize that both interventions (EMDR and KIDNET) are effective in reducing post-traumatic stress symptoms. Therefore, we do not expect (large) differences in effectiveness between the two interventions and hypothesize that both interventions equally reach efficacy in refugee children aged 8–18 years. However, this comparison has not been done before, in case there is a small difference in effectiveness, we aim to detect it.

## Methods

### Trial design

**Fig. 1:**
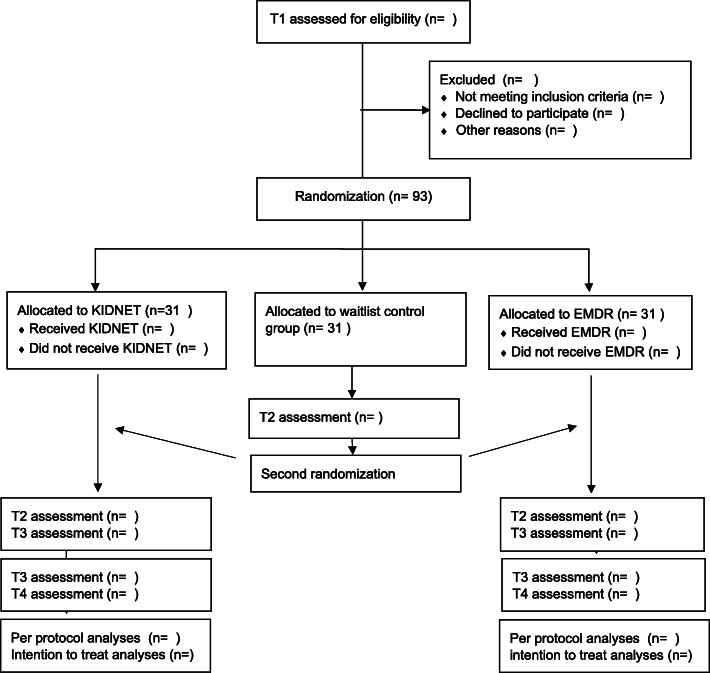
Adapted CONSORT flow diagram, illustrating the study design, the flow of participants, and the planned assessments

A multicenter, randomized controlled trial comparing KIDNET and EMDR to a waitlist control group at various locations in the Netherlands will be conducted. Refugee children (*N*=93) between 8 up to and including 18 years of age will be randomly assigned to either EMDR (*n*=31), KIDNET (*n*=31), or a waitlist control group (*n*=31). When initially assigned to a treatment condition, participants and one of their parents or caregivers will be assessed three times: at intake (T1; maximum of 4 weeks before the first treatment session), at T2 (4 weeks after the last session), and T3 (3 months after the last session). Participants initially assigned to the waitlist control group will be assessed four times: at intake (T1) and after the waitlist period of 8 weeks (T2). Hereafter, the second randomization takes place and participants will be assigned to EMDR or KIDNET. T3 will be administered 4 weeks after the last session and T4 3 months after the last session. In the period between the last treatment session and follow-up, no trauma-focused treatment will be offered. Figure 1 depicts the flowchart of the study.

### Study setting

Participants will be recruited through various channels, e.g., primary schools, high schools, international transitional classes in the Netherlands (ISK; a class for students between 12 and 18 who are recently residing in the Netherlands and speak little or no Dutch), general practitioners, centers for youth and family, and posts published through social media. Additionally, participants will be recruited in two outpatient clinics: (1) ARQ Centrum’45 (the Dutch national center for specialist diagnostics and treatment of people with (complex) psychotrauma complaints) and (2) I-psy Youth and Family (part of a specialist center in intercultural psychiatry). Assessments and treatment sessions will be carried out at various locations in the Netherlands; therapists travel to the living location or a location nearby the residency of the participants (and suitable for therapy, guaranteeing privacy for instance).

### Participants

#### Eligibility criteria

Eligible participants for this study must meet all following criteria: (1) between 8 and up to and including 18 years old and (2) partial or full PTSD diagnosis as reported by the child (interviewed with the Clinician-Administered PTSD Scale for DSM5 - Child/Adolescent Version (CAPS-CA-5; 36). Partial PTSD is defined as either fulfilling three of the four symptom clusters or one symptom present in each of the four symptom clusters. Participants will be included when they (3) are accompanied by at least one caregiver and (4) applied for asylum in the Netherlands or are residing in the Netherlands since January 2015 or later. A potential participant who meets any of the following criteria will be excluded from participation in this study: (1) estimated intelligence level (< 80), (2) acute interfering psychiatric disorder in need for treatment first, (4) brain damage, (5) acute threat of deportation or moving within intervention period, (6) anti-epileptic and neuro epileptic medication, or (7) current severe substance abuse.

### Therapists

All therapists are certified clinical psychologists, with a broad experience in treating refugee children and families and sufficient training in both active treatment conditions. Each therapist received a 4-day basic training on NET by official NET trainers and additionally must have completed a minimum of one full KIDNET treatment under supervision. In addition, each therapist giving treatment in this study received a 9-day training level 1 and level 2 according to the standards of EMDR Europe.

### Procedure

When a child is identified as a potential participant an introductory meeting is planned with the caregiver(s), the child, a member of the research team, and, if needed, an intercultural mediator. An Eritrean and Syrian intercultural mediator are part of the research team. In addition to translation/interpretation, it is expected that they may increase the trust of the participants by sharing their cultural background, can explain the study in their native language, and discuss stigma related to the use of mental healthcare. The second meeting, after obtaining informed consent, consists of further intake questions and collecting T1 data. When the CAPS-CA-5 has been administered and when the participant meets the inclusion criteria, a participant number is assigned. Then, the therapist is chosen based on availability. Hereafter, the participant will be randomly assigned to the EMDR condition, KIDNET condition, or the waitlist control group. In this way, randomization will occur across therapists. In case of a treatment condition (EMDR or KIDNET), the first session starts within 4 weeks after T1. In the case of a crisis, a child psychiatrist is available for consultation—as is a normal procedure during treatment.

#### Informed consent, randomization, and blinding

During the intake, the assessor explains the aim, method, benefits, and potential hazards of the study. The child and his/her legal guardian will then receive the informed consent (also available in Arabic and English). When the child (12 years and older) and his/her legal guardian agree to participate, the assessor obtains informed consent. When a child meets the inclusion criteria, an independent colleague of ARQ Centrum’45 is contacted and uses random allocation software for the random allocation of the participants to EMDR, KIDNET, or the waitlist control group. Random allocation is performed using a randomized block design with variable block sizes and an allocation ratio of 1:1:1. After the allocation is known, the independent colleague will inform the research coordinator. Hereafter, the research coordinator will inform the therapist about the allocation. Neither therapists nor study participants can be blinded to the treatment condition given the nature of the interventions. Independent members of the research team will administer T1–T4 assessments and remain blinded to the two treatment allocations (EMDR and KIDNET). Statistical analyzes will not start until the final assessment has been done. Hereafter, data analysts will no longer be blinded for the treatment allocation.

### Interventions

#### EMDR

The EMDR intervention will follow the Dutch translation of the EMDR protocol for children, consisting of the eight-phase protocol of Shapiro [36] (updated Dutch protocol [32, 37]). The phases are history taking, treatment planning, preparation, reprocessing, installation of a positive cognition, check for and processing any residual disturbing body sensations, positive closure, and evaluation. At the start of the first session, the child and caregiver(s) are present. This session consists of psychoeducation on PTSD and the rationale for trauma treatment, and the specifics of EMDR are explained shortly. Next, an inventory of possible target memories is made. From there on, up to seven sessions of EMDR are being offered aiming at the reduction of emotional disturbance associated with the most distressing traumatic memories and/or fear of images of what could have happened. The therapist starts with the target memory with the highest Subjective Unit of Distress (SUD) on a 1–10 scale. Standard eye movements are applied for bilateral stimulation, although alternative distractive tasks can be chosen as well. The intervention consists of up to eight weekly sessions of 75 min. Criteria for early termination are when all the memories listed on the case conceptualization do not evoke tension and/or the complaints have disappeared.

#### KIDNET

For KIDNET, a Dutch translation of the NET protocol adapted for children is followed [28, 38]. The therapist starts the first session with the child and caregiver(s). Psychoeducation on PTSD complaints and the indication for Narrative Exposure Therapy will be given to both the caregiver(s) and child. Furthermore, the specifics of KIDNET will be explained shortly. The therapist will then continue the session with the construction of the lifeline. A rope or a string will be placed on the floor/table representing the course of life of the child starting from birth. The other end of the rope/string is rolled up to indicate the future. Next, the child can lay stones and flowers on the lifeline in a chronological way representing fearful, shocking, and/or traumatic events (stones) and pleasant and meaningful moments (flowers), respectively. The lifeline always starts with a flower representing birth. In this session, the child will describe all the flowers and stones briefly and factually. The next sessions are devoted to following the lifeline chronologically, processing the traumatic events per session (and in some sessions also the pleasurable and meaningful events) through narrative exposure. Each session the therapist will write down the narrative belonging to the period discussed in that session (stone and/or flower). The therapist reads the narrative to the child in the subsequent session and asks the child if he or she wants to change anything. After the rereading, the narration is continued chronologically along the lifeline. The last session the therapist will read the complete narration to the child and will hand it as a document to the child. The intervention consists of up to 8 weekly sessions of 75 min. Criteria for early termination are when all the memories listed on the case conceptualization do not evoke tension and/or the complaints have disappeared.

#### Waitlist control group

The aim of the current trial is to evaluate the effectiveness of KIDNET and EMDR treatment. Therefore a waitlist control group is chosen as a comparator. Once trauma-focused treatment has been indicated, a child may be assigned to the waitlist control group. When a participant is randomized to the waitlist control group, he or she will receive no trauma treatment for 8 weeks. This implies there will be no contact (except in case of emergency) for 8 weeks before the post waitlist measurements (T2) and then the start of either one of the treatment conditions (second randomization).

#### Parental guidance

During the intervention period (both EMDR and KIDNET) caregivers will receive parental guidance of one to four sessions of one hour, one at the beginning, two during treatment, and one at the end. Parental sessions are aimed to support the child during their trauma-focused intervention. The sessions focus on how to deal with the symptoms of the child, how to support the child, and how to be emotionally available as a parent with respect for the child’s own space and time to process. The sessions with caregivers have a maximum duration of 75 min each. The sessions (in terms of duration, kind, and frequency) that caregivers receive will be registered.

#### Modifications and concomitant care

There are no planned intervention modifications and no planned circumstances whereby participants will be removed from the intervention by the trial investigators. Participants may withdraw from the study and intervention at any time. Trauma-focused treatment, other than the assigned condition, is not allowed during the study period. Other usual care for participants continues throughout the trial and will be monitored.

#### Treatment integrity and adherence

All sessions will be video- or, if the participant only consents with audio, audio-taped. Treatment fidelity will be checked by assessing videotaped (or audiotaping) sessions. Although it is expected that children will be able to communicate in the Dutch language, interpreters will be used when this is needed (e.g., for communication with caregivers). All therapists follow supervision sessions (once every 6 weeks), using video recordings of the sessions, from an accredited KIDNET supervisor and EMDR supervisor (CdR) for the duration of the study to optimize treatment adherence. If the therapist estimates that a limited number of extra sessions are needed to complete the trauma treatment, this can be decided during the supervision with the supervisor’s approval and will be noted.

#### Post-trial care

If a participant has residual complaints or another request for help during the last follow-up measure it will be discussed in our multidisciplinary team. In case of residual PTSD complaints, it will be discussed if the participant can receive additional trauma-focused treatment at ARQ Center’45 or a suitable referral to treatment nearby the living location of the participant will be made in co-operation with the referrer of the participant. The intervention is not expected to cause any harm.

### Measures

#### Timing of measurements

The Life Events Checklist-5 (LEC-5) will solely be administered at T1. The assessor will note important changes or events during the first and last measurement at the last measurement (T3 or T4). The *Clinician-Administered PTSD Scale for Children and Adolescents* (CAPS-CA-5 [39, 40]), the Children’s Revised Impact of Event Scale (CRIES-13 [41, 42]) parent and child version, the Strengths and Difficulties Questionnaire (SDQ [43]) parent and child version and the KIDSCREEN-27 [44] will be administered at each timepoint. Lastly, the CRIES-13 child version will be administered at each session by the therapist during the intervention.

#### Primary outcomes

##### Posttraumatic stress disorder and symptoms

At baseline, the LEC-5 will be administered to register the number and type of traumatic events the child was exposed to. To assess the presence of a (partial) PTSD diagnosis and symptom severity the CAPS-CA-5 for DSM5 will be administered. The CAPS-CA-5 is a 30-item structured clinical interview assessing PTSD symptoms based upon DSM5 criteria for children and adolescents aged seven and above. Symptoms in relation to the past month are examined. Symptom severity ratings are based on symptom frequency and intensity, with a severity score of two or higher indicating the presence of a symptom. CAPS-CA-5 can be administered reliably by different interviewers, with a kappa coefficient of .75 for the Dutch (DSM-IV) version [45] and .80 for the English DSM5 version [46]. The Dutch CAPS-5 is a carefully translated instrument with adequate psychometric properties [47].

Furthermore, both the child version and the parent version of the CRIES-13 will be administered. The CRIES-13 is a brief self-report measure to screen children aged 8 years and above for the severity of PTSD symptoms. The CRIES-13 consists of 13 items to assess intrusion, avoidance, and hyperarousal. The CRIES-13 child version has demonstrated good reliability among war-affected children and adolescents [42]. A study evaluating the reliability and validity of the CRIES-13 child version in a large clinically referred sample in the Netherlands (*n*=395) reported an internal consistency using Cronbach’s *α* of .89 and a test-retest reliability coefficient of .85 [48].

#### Secondary outcomes

##### Behavioral and emotional symptoms

The SDQ is a brief behavioral screening questionnaire that measures emotional symptoms, behavioral problems, hyperactivity, peer relation problems, and prosocial behavior. Both child and caregiver versions are administered.

##### Quality of life

The KIDSCREEN-27 (child version) is a generic quality of life measure based on physical well-being, mental well-being, autonomy, relationship with parents, friendships, and functioning at school. The KIDSCREEN-27 has been designed and normed for children and adolescents aged 8–18 years.

##### Demographic variables

A social demographic form will be filled in by the assessor based on the intake interview to obtain information on age, gender, country of birth, asylum status, time in the Netherlands, and whether the child and/or parents need(s) a translator.

##### Important changes or events between Time 1 and Time 3/Time 4

A form with several questions about important events or changes during the first and last measurement will be filled in by the assessor. Examples are changes in asylum status, change of location within the Netherlands, and changes in the family situation (divorce or reunification).

#### Translation of instruments

All self-report questionnaires, except the KIDSCREEN-27, for which no validated translations were available, have been translated—and subsequently back-translated by professional bi-lingual interpreters. Because they are spoken by relatively large numbers of refugees in the Netherlands, Tigrinya and Arabic have been chosen for translation. The assessments of participants, speaking a language other than Tigrinya or Arabic and when an officially back-translated questionnaire is not available in their language, will be done in Dutch and translated on the spot by a telephone interpreter.

#### Sample size

Previous studies with refugee children show large between-group effect sizes for PTSD symptom severity for KIDNET vs control group and EMDR vs control group [18, 20, 21, 25]. Therefore, at least a medium effect size can be expected when comparing the two treatment conditions (EMDR and KIDNET) to the waitlist control group. With the following assumptions: a correlation between assessments of 0.40, a modest effect size of intervention (ES *f*=0.20), a power of .80, alpha of .05, and two groups (EMDR, KIDNET versus waitlist control group), a total sample of 78 participants will be needed (26 participants per condition), (G*power [49]).

We hypothesized that both interventions (EMDR and KIDNET) are effective in reducing post-traumatic stress symptoms. In case there is a small difference in effectiveness, we aim to detect it. Therefore, the number of participants needed to detect a small effect size (*f*=0.17) is computed, resulting in a preferable sample size of 78 participants (39 participants per treatment condition; EMDR and KIDNET).

After the waitlist period of 8 weeks, the second randomization will take place. Participants will be randomly assigned to EMDR or KIDNET which results in 13 extra participants in both treatments group and thereby a total of 39 participants in both treatment groups. Since a crossover design is not used, the data of the participants (*n*=26) who were secondly randomized into a treatment condition will only be used in the analyses of EMDR versus KIDNET. A conservative 20% attrition rate will be taken into account, resulting in a total required sample of 93 participants.

### Statistical analyses

Descriptive statistics and analysis of variance will be performed. All three groups will be compared with regard to pre-treatment variables such as demographic variables (age, gender, ethnicity). To test the effectiveness of EMDR vs waitlist control group and KIDNET vs waitlist control group, analyses of variance will be performed with time (T1 and T2) as within-subject variable, and treatment (EMDR, KIDNET, or waitlist control group) as between-subject variable. To test the effectiveness of EMDR compared to KIDNET, analyses of variance will be performed with time (pre-treatment, post-treatment, and follow-up) as within-subject variable, and treatment (EMDR and KIDNET) as between-subject variable. Both analyses will be done twice; with per-protocol sets and intention-to-treat sets. SPSS will be used for multiple imputations techniques for missing data, the per-protocol analyses, and the intention-to-treat analyses. CONSORT criteria will be followed for the statistical analyses (and reporting) of randomized controlled trials. Repeated measures (per session) will be conducted to evaluate treatment progress (CRIES-13 child version).

### Data collection and management

Assessors have received a one-day training in administering the CAPS-CA-5. To promote participant retention and complete follow-up, participants are given a gift card worth fifteen euros per assessment for completing the T2, T3, and if applicable T4 assessments. The same assessments will be planned in the case of deviations from the protocol. In addition, an attempt is made to plan the same assessments for participants who discontinue treatment. Data will be handled confidentially and stored anonymously. A subject identification code will be used to link the research data to the subject. The key to the code will be kept separate by the principal investigator. The handling of personal data will be according to the Dutch Personal Data Protection Act (in Dutch: De Wet Bescherming Persoonsgegevens, Wbp).

### Project organization

The Trial Steering team is meeting three times a year to assure protocol adherence, oversee conduct and process as well as to exchange their expertise about running an RCT. This team consists of professors and health care professionals, among which one expert on KIDNET, one expert on EMDR applied to children, one expert on the CAPS-CA-5, and a colleague from I-psy Youth and Family. In addition, there is one team providing day-to-day support for the trial. This team consists of six people (one professor/clinical psychologist, two cultural mediators, two assessors, one PhD student/assessor). This team is meeting once every 2 weeks and is responsible for the assessments, local organization, recruitment of participants, assessments, and taking informed consent.

### Monitoring

No interim analyses will be performed and there are no stopping guidelines. In case of protocol amendments, an addendum will be sent to the Medical Ethical Committee (METC) and the funders will be noted and asked for permission.

### Dissemination plans

The main findings of this study will be disseminated via publications in peer-reviewed international journals. Presentations of study findings will also be offered at relevant research conferences, and local academic symposia and seminars.

## Discussion

As the number of refugees has increased dramatically in the past decade, and half of its population is below the age of 19 years, there is a high need for effective interventions aimed to counteract mental health difficulties. Repeatedly and consistently, refugee children are at increased risk for mental health problems in particular posttraumatic stress (symptoms as well as disorder). Yet negative mental health consequences of experiences with war, violence, and disruption are reluctantly acknowledged and refugees are for several reasons not inclined to seek mental health care, even when this is available. This paper describes a protocol of a randomized controlled trial in refugee children 8–18 years, who reside with at least one caregiver, in the Netherlands. Two trauma-focused interventions are offered at a location close to the living location of the child and his/her caregiver(s). Consort criteria guide the methodology of the study. A total of 93 children of various origins will be recruited for participation. The aim of the study is to determine the efficacy of two evidence-based trauma-focused interventions, KIDNET and EMDR, both compared to a waitlist control group and with each other.

### Risks

Risks may be related to the recruitment of participants. In order to gain trust and to be able to effectively communicate the aims and procedures of the project, intercultural mediators are part of the team. Coming from Eritrea and Syria themselves, they share the background of the target populations. They will be able to reach out to parents and adolescents, speaking Tigrinya and Arabic, and are also able to connect to other groups. There is a risk of recruitment difficulties due to restrictions because of the COVID-19 pandemic. Fortunately, meetings of two people (from different households) are still allowed in the Netherlands.

### Generalizability of findings

Measures that we use have demonstrated psychometric qualities in previous studies, and at least some have shown cross-cultural validity. Still, the instruments are based on Western constructs, and although procedures for translation and back-translation have been followed, and interpreters will be involved whenever needed (for instance in meeting with the parent(s)), culturally determined interpretations may cause bias. Each assessment will be conducted by an independent assessor who will carefully monitor observations of the encounter. Besides, we will monitor and determine treatment fidelity. These data may direct later strategies for implementation of interventions if these are deemed feasible and effective.

In conclusion, this is the first randomized controlled trial focusing on two types of trauma-focused treatment for refugee minors with PTSD, compared to a control group. The ultimate goal is to increase the accessibility of PTSD treatment for refugee minors when it is efficacious.

## Trial status and protocol version

Recruitment started on 15-03-2018 and will presumptively end on 30 June 2022

Protocol version number: 2

Protocol version date: submitted on 11th of March 2022

## Data Availability

Due to the nature of this research, participants of this study did not give consent for their data to be shared publicly, so supporting data is not available.

## Abbreviations

PTSD: Posttraumatic stress disorder
EMDR: Eye Movement Desensitization Reprocessing
KIDNET: Child-focused Narrative Exposure Therapy
RCT: Randomized controlled trial
NET: Narrative Exposure Therapy
TAU: Treatment as usual
LEC: Life Events Checklist-5
CAPS-CA-5: Clinician-Administered PTSD Scale for Children and Adolescents for DSM5
CRIES-13: Children’s Revised Impact of Event Scale
SDQ: Strengths and Difficulties Questionnaire
METC: Medical Ethical Committee
